# Extirpation of the Larynx

**Published:** 1882-01

**Authors:** 


### EXTIRPATION OF THE LARYNX.

Professor A. Ceccherelli has published recently (*Lyon Medical*) a short account of the operations for complete or partial extirpation of the larynx performed up till now, with a statement of the results obtained. The operations included in the list amount to thirty in number, of which twenty-five were cases of total extirpation, and five cases of partial resection. The operation has been performed eighteen times for a carcinoma. In twelve other cases, the tumor was either not of a malignant nature, or the nature of the lesion for which the operation was undertaken was not recognized. Of these thirty cases, cure is said to have been obtained twenty times; and in the twenty-first case, the result is unknown. Twenty of the individuals, however, who recovered from the operation, succumbed later with symptoms of early relapse. The final result, then, shows eleven undoubted cures, or, at least, considerable prolongation of life, and one doubtful case. Professor Ceccherelli's conclusions are the following: 1. Extirpation of the larynx is an operation henceforth included in surgical therapeutics; 2. It is best carried out by the thermo cautery; 3. It should be reserved for those cases in which the lesion is not too diffused for the successful attack of the surgical knife; 4. Tumors of a malignant nature are not suitable to the operation; 5. The operation is very admissible in cases of carcinoma, but only when it is limited, and when the case is not one of soft cancer, and the disease has not yet produced general inflammation.—*Med. and Surg. Reporter.*

